# Setting the basis for a natural language processing tool to improve the reporting of notifiable diseases: a descriptive study

**DOI:** 10.3389/fdgth.2026.1882377

**Published:** 2026-10-20

**Authors:** Clarisa Cano-Escobar, Lourdes Estefanía Barón-Miras, Juan José Gualda-Gea, Santiago Frid, Maria Jesús Bertran

**Affiliations:** 1 Faculty of Medicine and Health Sciences, Universitat de Barcelona, Barcelona, Spain; 2 Preventive Medicine and Epidemiology Department, Hospital Clínic de Barcelona, Barcelona, Spain; 3 Instituto de Salud Global de Barcelona (ISGlobal), Barcelona, Spain; 4 Clinical Informatics Service, Hospital Clínic de Barcelona, Barcelona, Spain

**Keywords:** electronic health records, epidemiologic surveillance, natural language processing, notifiable diseases, public health

## Abstract

Timely reporting of notifiable diseases is crucial for public health, but it is often delayed by semimanual International Statistical Classification of Diseases and Related Health Problems Tenth Revision (ICD-10) coding of electronic health records (EHRs). Natural language processing (NLP) could enhance timely reporting by identifying potential cases of patients in unstructured clinical documents. This study aimed to describe the clinical documents containing key terms that suggest notifiable diseases for informing the future development of an NLP pipeline in a tertiary hospital, and this study also aimed to evaluate the current semimanual hospital notification system. This retrospective observational study included all the notifiable disease episodes according to Catalonian regulations reported between May and July 2023 from a tertiary hospital in Barcelona. Information on age, sex, notification-related variables, and the healthcare area where suspicion was determined was obtained from the hospital surveillance database. The adequacy of disease suspicion on the reporting date was assessed based on the regional public health criteria. For correct and timely notifications, the frequency of the presence of clinical documents and the frequency of their inclusion of terms that raise suspicion were described. Notification delay since the first date of evidence of suspicion in the EHR was calculated. Results were presented globally and stratified by 10 disease groups, with eight being communicable disease groups. A total of 284 episodes were notified. The most frequent group was sexually transmitted diseases (25.8%), with the outpatient clinic being the healthcare area where most notifications were found (145, 51.2%). Adequate notifications accounted for 210 (74.2%) episodes, including 25 diseases. Incorrect notifications were obtained mainly due to the reporting of chronic conditions as onset diseases (34.2%). Up to 25% of notifications were delayed for 3 days or more. Terms that raise suspicion were mostly found in medical notes (93.0%–78.7%). The unreleased clinical draft, the clinical course, and the microbiology reports were available for over 50% of the episodes of six or more disease groups, with a high inclusion of such suspicion-raising terms (81.1%–94.8%). The results revealed suboptimal reliability and faster reporting in the current reporting process. As the goal was to identify notifiable diseases before the confirmation of diagnosis, suspicion-raising terms were frequently found in unstructured clinical notes. Hence, the integration of the described documents into an NLP-based workflow could improve the reporting process.

## Highlights

- Natural language processing (NLP) could support reporting of notifiable diseases by identifying potential cases of patients upon suspicion, particularly within unstructured clinical documents.
- This study found that, within the semimanual reporting system of a tertiary hospital, up to 25% of notifications were delayed by 3 days or more and 25.8% of notifications were classified as inadequate.
- In addition to hospital and emergency room discharge notes, other documents—such as unreleased clinical drafts and clinical course notes—were extensively present in notifiable disease episodes, with a high inclusion of suspicion-raising terms. Therefore, the inclusion of these documents into an NLP-based workflow could improve the reporting process.

## Introduction

Notifiable diseases are, as defined by the WHO International Health Regulations (IHR) of 2005, “all those events which may constitute a public health emergency of international concern” and are therefore susceptible to reporting to the pertinent public health authorities to determine and provide the required measures to rapidly prevent the transmission of such diseases (1, 2). The diseases comprehended under the scope of this definition vary between countries, and although they mainly comprise infectious diseases, some non-communicable entities of epidemiological interest may also be included. The WHO proposes a decision algorithm that summarizes which events shall be notified (1).

The key to a successful response to a public health emergency is the early detection and reporting of events. The notification process and its particularities are designed by every state party of the WHO in accordance with the IHR stipulations. In the case of Spain, the list of notifiable diseases and the epidemiological surveillance structure follow the guidelines of the European Union (Directive 2003/99/EC and further updates) (3–5) in accordance with the aforementioned IHR, and further organizational concretions are regulated by each autonomous community (6–9).

Although the intricacies in the reporting process are firmly established, currently, there is no standardized surveillance protocol. In addition, although treating physicians are expected to report notifiable diseases to the pertinent authorities upon suspicion (8), it has been widely suggested that a significant underreporting exists among them. Studies propose the concurrence of multiple factors as an explanation to this phenomenon (e.g., poor knowledge of the conditions subject to notification, ignorance of the procedure, lack of time and motivation, or mere incompliance) (2, 10, 11). Therefore, it is common practice to establish a notification team to perform or coordinate this task (12). However, these teams frequently lack appropriate informatics tools to detect potential episodes with a notifiable disease and therefore rely on manual reviewing. Previous studies have suggested that, under such surveillance systems, reporting may not only be subject to human mistakes derived from the overseeing of the indices of a disease or by their misjudgment in non-trained professionals (12), but it can also be delayed given the fact that it depends almost entirely on their periodical surveillance. Therefore, holidays and weekends, incomplete review of episodes, or increased workload in other areas of the daily practice may lead to a delayed notification.

The implementation of information technologies in medical practice has significantly enhanced the accessibility and efficient management of patient data. Codified clinical terminology systems such as Systematized Nomenclature of Medicine—Clinical Terms (SNOMED CT) and International Statistical Classification of Diseases and Related Health Problems, Tenth Revision (ICD-10) have allowed the standardization of terms and a systematic gathering of clinical data, as well as their successful communication and analysis. They also set the basis for clinical decision support systems (13–15). These tools tend to rely on structured information within electronic health records (EHR). Yet, healthcare workers tend to write long, unstructured clinical notes that allow for a more granular, less restrained narrative description of the clinical status and progression of the condition of patients. However, this approach makes it difficult to reuse clinical data, given the lack of capacity of IT systems to understand the semantics of the clinical concepts in free texts. Natural language processing (NLP) is a technique within the sphere of artificial intelligence (AI) that is designed to handle free text or speech data. It can successfully understand complex uncoded natural language and infer an output based on the training received. In medicine, NLP has innumerous applications: from information extraction for basic research to the development of interactive patient-facing platforms (16). In the field that concerns this study, compared with human review, NLP has demonstrated high sensibility, specificity, and positive predictive value in the identification of notifiable conditions, directly extracting a given term from the free text present in laboratory records (17). It has also shown a clear potential to minimize costs and delays while improving patient safety when suggesting diagnoses from free-text EHR notes (18). This, along with the fact that AI can work on larger datasets and dynamically learn in a shorter timeframe than humans, turns NLP into a valuable tool for identifying notifiable diseases.

Hospital Clínic de Barcelona (HCB) has implemented tools within its health information system that detect ICD-10 codes recorded in the EHR and map them to their corresponding notifiable disease codes (required by the Public Health Agency of Barcelona). Because many of the diagnoses are not immediately coded and many terms that lead to suspicion are not granular enough, this process reveals itself as suboptimal, making human review indispensable. Regarding NLP, the hospital has a tool that recognizes diagnoses and procedures only in free text in discharge notes (and not in other documents of the EHR) and assigns them their corresponding ICD-10 codes, which are later reviewed by clinical documentation specialists. However, the current use of this NLP tool does not allow the early identification of most notifiable conditions in patients because this process occurs within the month following their discharge.

## Objectives

This study aimed to describe the clinical documents from Hospital Clínic de Barcelona containing key terms that suggest notifiable diseases and to evaluate the current reporting process to set the basis for the development of an NLP tool in the field of disease notification.

To fulfill this purpose, two specific objectives were set. The first one was to describe the viability of detecting clinical entities of different notifiable diseases in the predefined clinical documents. The second one was to evaluate the current reporting methodology and to identify aspects that improvement.

## Methods

Figure 1 describes the methodological workflow. This retrospective observational study included all episodes of notifiable diseases under Catalonian regulations (6) reported by Hospital Clínic de Barcelona, a tertiary hospital in Spain, between May 2023 and July 2023. No notifiable condition, communicable or non-communicable, was excluded, and therefore, the conditions analyzed correspond to those actually notified during the study period rather than to a predefined selection. The study period was chosen because summer is the time of year when some notifiable diseases, such as dengue, Zika, and chikungunya, are most frequently reported in Catalonia, which allowed capturing of a broader range of notifiable conditions, and these conditions are listed in Table 1. As the unit of analysis is the notification episode, one unique patient could have more than one episode of notifiable disease. Data on age, sex, disease, the epidemiological week of report, the date of the actual report, and the healthcare area where the suspicion arose—hospital (including home hospitalization episodes), emergency room (ER), or outpatient clinic—were collected. The “adequacy of the suspicion” was assessed, which was defined as the existence of sufficient data on the date of the actual report at the reach of the notifiers that justified the notification. Potential causes that led to incorrect reporting were classified into five categories: (1) “chronic”: patients that had one chronic disease among the notifiable diseases that was diagnosed only in a past episode and supposedly already reported; (2) “history”: patients in whom the notifiable disease is an element of the past pathological history; (3) “context”: cases of patients with documents containing synonymous terms that are not sufficient to raise suspicion of a notifiable disease; (4) “no data”: cases of patients with no documents available or with documents with suspicious terms; and (5) “mismatch”: patients with documents that were reported a certain notifiable disease but, when they presented the terms, led to the suspicion of a different notifiable disease. This assessment was performed by medical doctors of the investigation team. When consensus was not reached or doubts persisted, the more experienced epidemiologists decided the result. The case definitions and notification criteria of the Public Health Agency of Catalonia were applied to the information available in the EHR up until the notification date.

**Figure 1:**
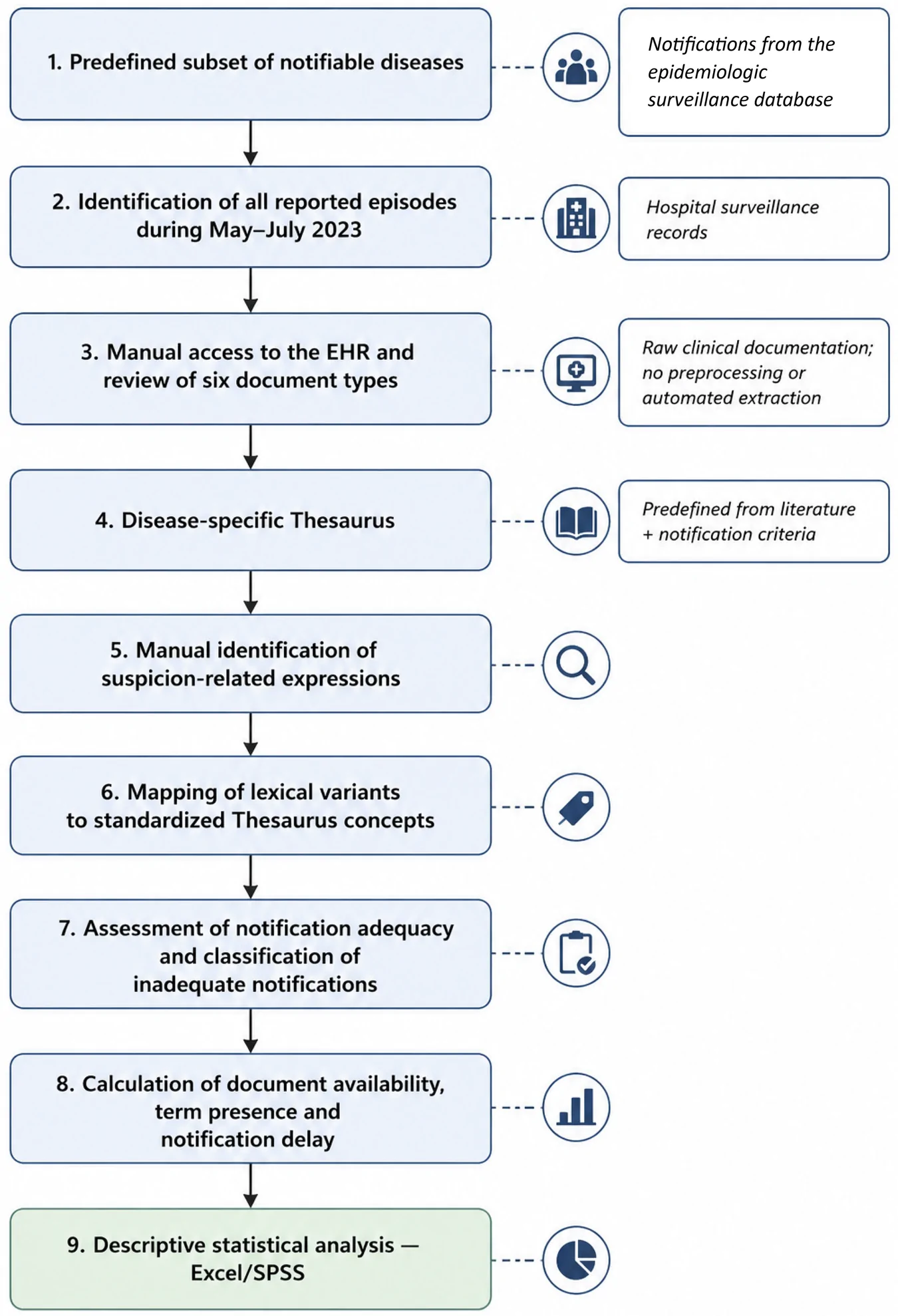
Methodological workflow.

**Table 1:** Classification and distribution of the notified episodes between May 2023 and July 2023 by disease groups in the Hospital Clinic of Barcelona.

Disease	Number of patients	Disease group	Disease category	Number of patients
Asthma	57	Asthma	Non-communicable	57
Chickenpox	6	Exanthematous viral diseases and mumps	Communicable	9
Parotiditis	3		Communicable	
Shigellosis	4	Foodborne and waterborne diseases	Communicable	6
Typhoid and paratyphoid fever	2		Communicable	
Heatstroke	12	Heatstroke	Non-communicable	12
Hepatitis B	19	Hepatitis	Communicable	44
Hepatitis C	25		Communicable	
Meningococcal disease	2	Invasive bacterial diseases of urgent notification	Communicable	2
Chikungunya	1		Communicable	
Chikungunya virus disease	2		Communicable	
Dengue	29	Mosquito-borne diseases	Communicable	40
Malaria	7		Communicable	
Zika	1		Communicable	
Hydatidosis	3		Communicable	
Leishmaniasis	4	Other zoonoses	Communicable	9
Leptospirosis	2		Communicable	
Other acute respiratory	2		Communicable	
Legionellosis	6	Respiratory infectious diseases	Communicable	31
Tuberculosis	23		Communicable	
Acquired immunodeficiency syndrome (AIDS)	1		Communicable	
Chlamydia genital infection	4	Sexually transmitted diseases	Communicable	73
Gonococcal infection	28		Communicable	
Syphilis	25		Communicable	
VIH infection	15		Communicable	
Total	283			283

Hospital Clínic de Barcelona is predominantly an adult tertiary hospital and does not provide general pediatric care, with pediatric activity limited to neonatology.

Regarding the identification of the specific terms alluding to each disease, a thesaurus compiling them was developed prior to data analysis. Its confection was grounded in the bibliographic research of the clinical characteristics and the natural history of each notifiable entity along with the consideration of the regulations governing the reporting process. Subsequently, clinical documents were manually reviewed by the investigators in their original form. No automated extraction, NLP procedure, text cleaning, spelling correction, normalization, or removal of content was performed before or during document assessment. Thus, abbreviations, typographical errors, idiomatic expressions, colloquialisms, and other forms of linguistic variability were retained as documented in the EHR. When different lexical forms were judged to refer to the same clinical concept, they were mapped after identification to a standardized thesaurus entry—in Catalan, lowercase, and without accents—solely for classification and quantitative analysis. Therefore, standardization was applicable for the representation of the identified concepts and not to the source clinical text. The presence of these concepts was assessed in the following clinical documents: (1) the emergency room discharge note, (2) the hospital discharge note, (3) the unreleased clinical draft, (4) the clinical course report, (5) the laboratory report, and (6) the microbiology report. The selection of these documents was based on the perspective of the Preventive Medicine and Epidemiology Department upon their consultation regarding the most relevant and informative documents in the EHR. The presence of suspicion was determined by quantifying the number of documents containing keywords from the thesaurus in both the adequate and inadequate reports. In adequate reports, the presence of terms that raise suspicion was described for each included document by their healthcare area and disease group (asthma, exanthematous viral diseases and mumps, foodborne and waterborne diseases, heatstroke, hepatitis, invasive bacterial diseases of urgent notification, mosquito-borne diseases, other zoonoses, respiratory infectious diseases, and sexually transmitted diseases [STDs]).

Data treatment and analysis were performed using Microsoft Excel and the software platform IBM® SPSS Statistics 29.0.0.0.

## Results

A total of 284 episodes comprising 110 females, 173 males, and 1 person of unknown sex was analyzed. The median age was 42 years (interquartile range [IQR]: 25). A total of 25 different notifiable diseases were observed in the sample, which were then categorized into 10 disease groups, as shown in Table 1. The most frequent group was sexually transmitted diseases (25.8%). A demographics summary of the sample is presented in Table 2. One patient was excluded from further analyses since they lacked information of any kind in the clinical records, leaving a total of 283 individuals subject to study: 145 (51.2%) from the outpatient clinic, 96 (33.9%) from the emergency room, and 42 (14.8%) from the hospital.

**Table 2:** Demographics summary of the notified episodes by disease groups between May and July 2023 by disease groups in the Hospital Clinic of Barcelona.

Disease group	Age	Sex, *n* (%)		Area, *n* (%)			Total (283), *n* (%)
		Female	Male	ER^a^	Hospital	Outpatient clinic	
Asthma	50.3 (±21.3)	38 (67)	19 (33)	39 (68.4)	18 (31.6)	0	57 (20.1)
Exanthematous viral diseases and mumps	41.8 (±22.5)	3 (33)	6 (67)	8 (89)	1 (11)	0	9 (3.1)
Foodborne and waterborne diseases	41.3 (±17.8)	1 (17)	5 (83)	2 (33.3)	2 (33.3)	2 (33.3)	6 (2.1)
Heatstroke	54.2 (±26.1)	7 (58)	5 (42)	12 (100)	0	0	12 (4.2)
Hepatitis	52.2 (±14.1)	15 (34)	29 (66)	0	2 (4.5)	42 (95.5)	44 (15.5)
Invasive bacterial diseases of urgent notification	48.5 (±30.4)	2 (100)	0	1 (50)	1 (50)	0	2 (0.7)
Mosquito-borne diseases	37.7 (±10)	20 (50)	20 (50)	12 (30)	1 (2.5)	27 (67.5)	40 (14.1)
Other zoonoses	58.2 (±19.6)	5 (56)	4 (44)	0	0	9 (100)	9 (3.1)
Respiratory infectious diseases	48.7 (±20.1)	6 (19.4)	25 (80.6)	7 (22.5	8 (25.8)	16 (51.6)	31 (11)
Sexually transmitted diseases	36.8 (±11.9)	12 (16.4)^b^	60 (82.1)^b^	15 (20.5)	9 (12.3)	49 (67.1)	73 (25.8)

a ER, emergency room.

b For the “sexually transmitted diseases group,” one of the episodes reported was from a patient of unknown sex.

The notification of the suspicion was adequate in 210 (74.2%) patients and incorrect in 73 (25.8%) of them. Among the correctly suspected ones, up to 170 were confirmed to have the reported condition. Thirty-three patients (15%) were reported without an explanation before there was a suspicion in the EHR, and therefore, they were excluded from further analysis. The delay for the remaining 177 patients had a median of 0 days (IQR: 3 days). Up to 62% of these patients were reported on the same date that the first suspicion came up in the EHR. However, 10% was reported later than 2 weeks after the first suspicion.

### Characteristics of inadequate notifications

The most frequent reason for an inadequate notification was the identification of a chronic condition as a new reportable event (34.2%), followed by references to a disease in the patient's medical history (26.0%) and disease-related terms whose clinical context did not support the current suspicion (23.3%). The absence of supporting information accounted for 12.3% of inadequate notifications, while 4.1% of notifications involved a mismatch between the disease reported and the disease suggested based on the available information (Figure 2).

**Figure 2:**
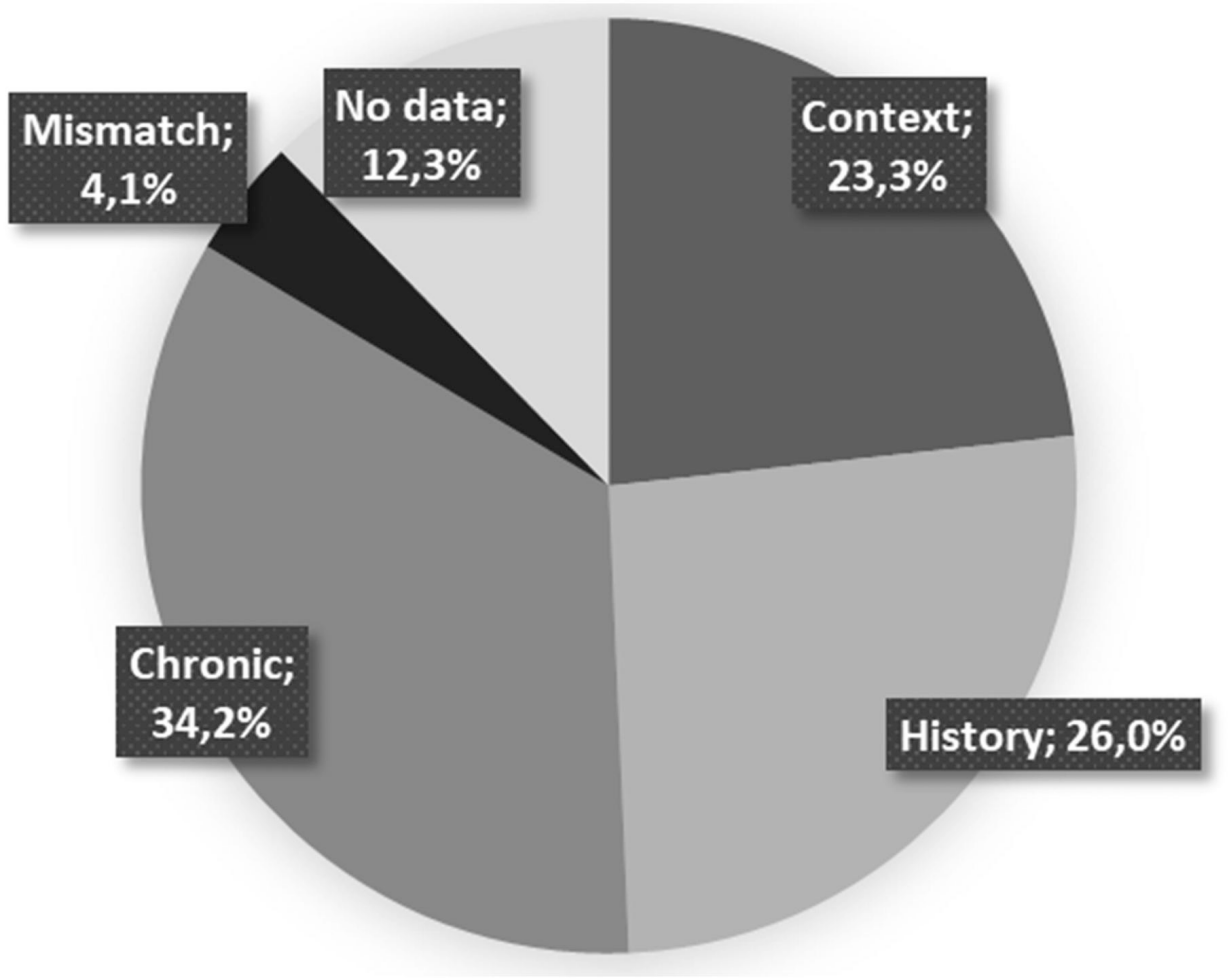
Distribution of causes for mistaken suspicion in notified episodes between May 2023 and July 2023 by disease groups in the Hospital Clinic of Barcelona.

Notably, 83% of inadequate notifications contained disease-related terms included in the thesaurus but lacked sufficient contextual evidence to support notification of the reported condition. Thus, the presence of a disease-related term alone did not consistently correspond to an adequate disease suspicion.

### Distribution of suspicion information across EHR document types

Among adequately notified episodes with identifiable supporting information in the EHR, suspicion-related terms were found across all the six document types evaluated. When the corresponding document was available, these terms were identified in 93.0% of hospital discharge notes, 89.0% of unreleased clinical drafts, 84.7% of ER discharge notes, 78.7% of clinical course reports, 63.3% of microbiology reports, and 45.4% of laboratory reports.

However, document availability differed substantially according to the healthcare setting and the disease group. Consequently, a high proportion of suspicion-containing documents did not necessarily indicate that a document represented a frequent source of information across the overall cohort.

### Variation by healthcare setting

The distribution of suspicion-related information differed across healthcare settings (Figure 3). In ER episodes, ER discharge notes and unreleased clinical drafts were the predominant sources, containing suspicion-related information in 85.4% and 83.8% of all ER episodes, respectively. Clinical course reports contributed less frequently in this setting, while hospital discharge notes and microbiology reports were often unavailable.

**Figure 3:**
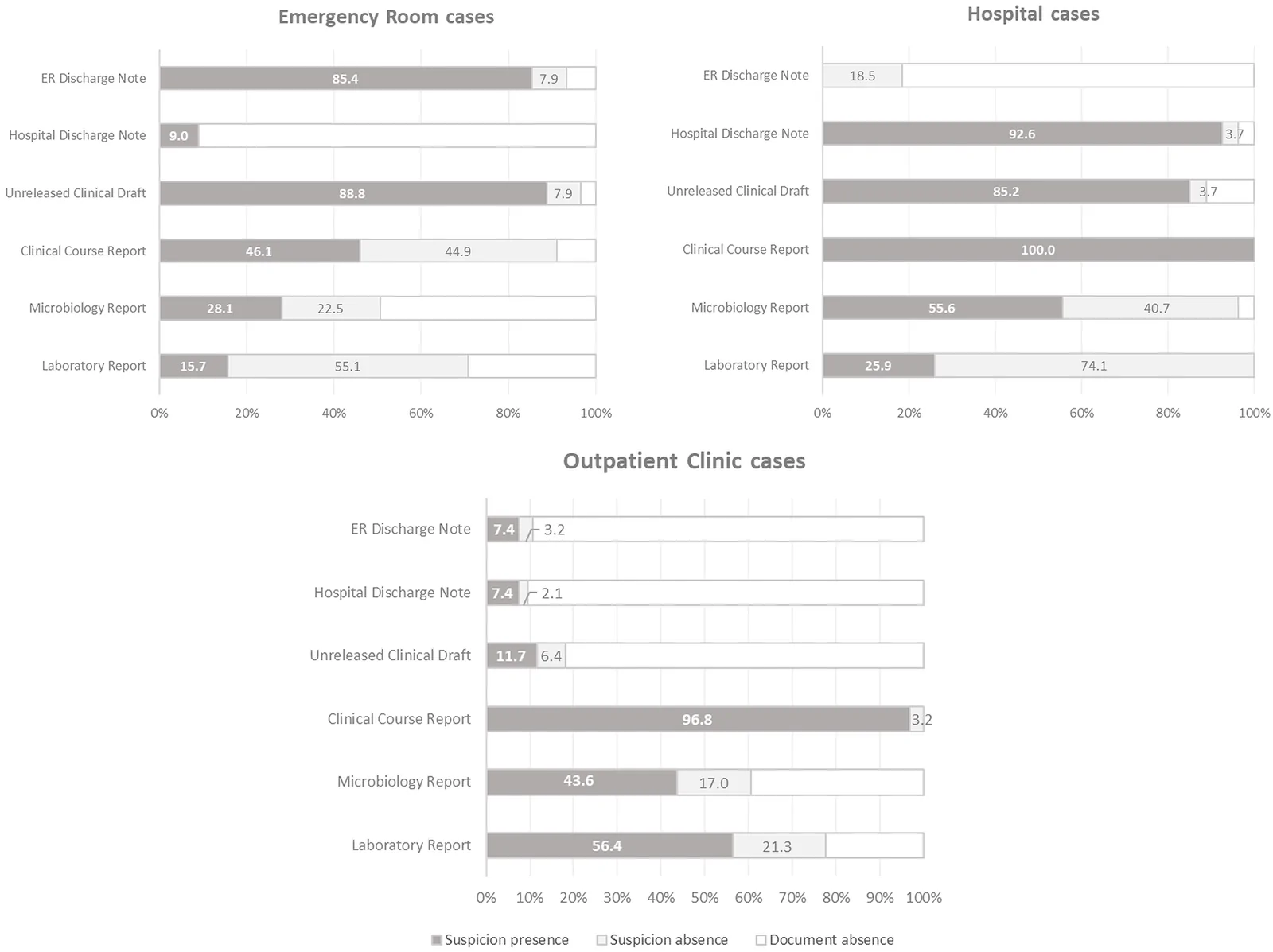
The presence of suspicion terms of a notifiable disease in the different document types by healthcare area in notified episodes between May 2023 and July 2023 by disease groups in the Hospital Clinic of Barcelona.

Among hospitalized patients, clinical course reports contained suspicion-related information in all episodes for which they were assessed, and these reports were accompanied by a high contribution from hospital discharge notes and unreleased clinical drafts. Conversely, ER discharge notes were uncommon in this setting.

In the outpatient clinic, clinical course reports were the predominant source of suspicion-related information, containing relevant terms in 96.8% of episodes. Laboratory and microbiology reports also contributed information, whereas ER discharge notes, hospital discharge notes, and unreleased clinical drafts were not frequently available.

Overall, the document types containing suspicion-related information were therefore strongly dependent on the healthcare setting in which the episode was managed.

### Variation by disease group

The contribution of each document type also varied across disease groups (Figure 4). The clinical course report was the most consistently available document, which was available in more than 80% of episodes in every disease group, and contained suspicion-related terms in 81.2% of the episodes for which it was available.

**Figure 4:**
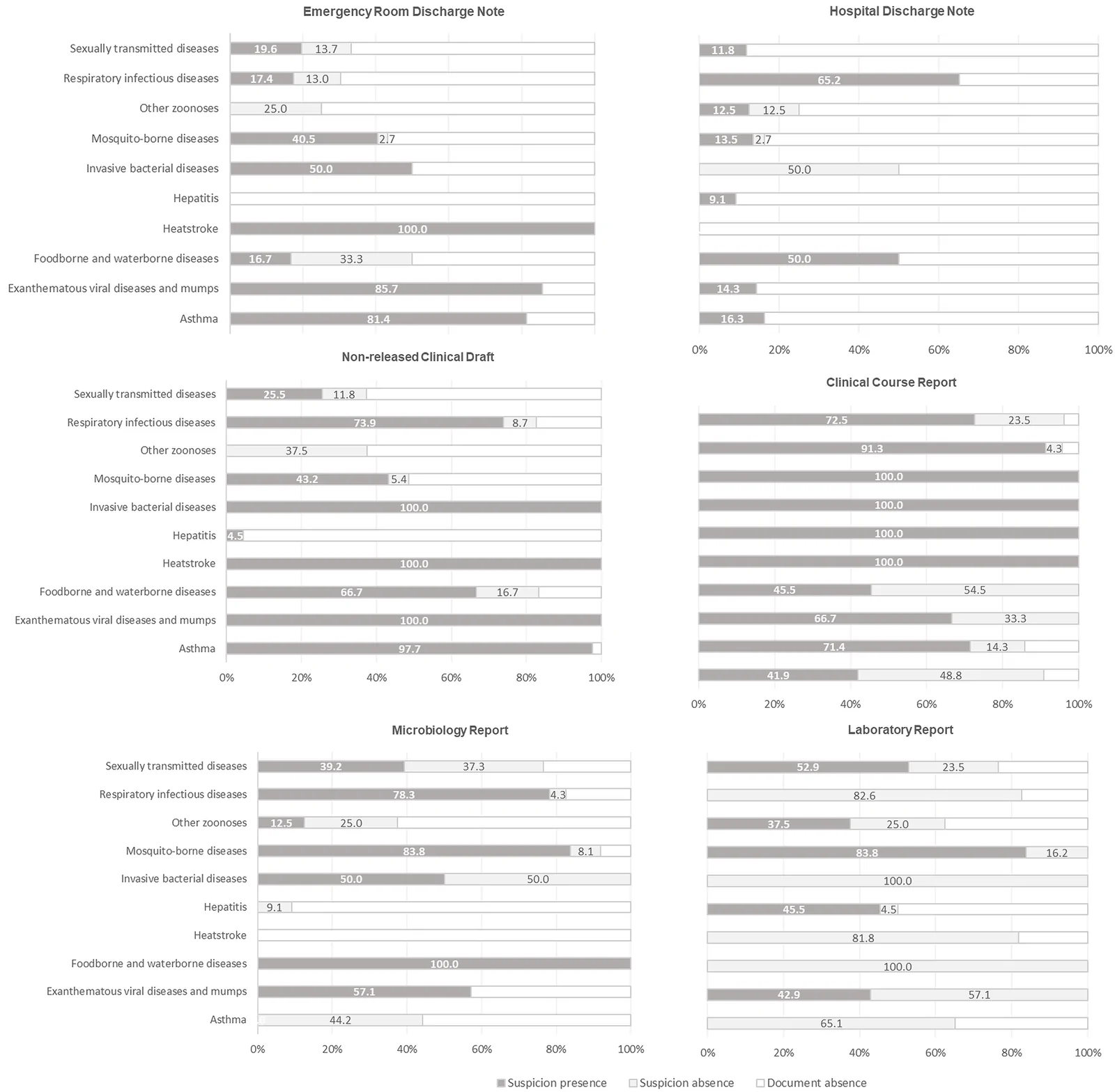
The presence of suspicion terms of a notifiable disease by healthcare area and disease group in notified episodes between May 2023 and July 2023 by disease groups in the Hospital Clinic of Barcelona.

Unreleased clinical drafts were representative—defined as being present in more than 50% of episodes—in six disease groups and contained suspicion-related terms in 94.8% of the available documents within these groups. ER discharge notes were representative primarily for asthma, exanthematous viral diseases and mumps, and heatstroke, whereas hospital discharge notes were representative only for respiratory infectious diseases.

The contribution of diagnostic reports was more disease-dependent. Microbiology reports were representative in six disease groups and, when available within these groups, contained suspicion-related terms in 81.1% of episodes. Particularly high proportions were observed for exanthematous viral diseases and mumps, foodborne and waterborne diseases, mosquito-borne diseases, and sexually transmitted diseases. Laboratory reports showed greater variability overall, although high proportions of suspicion-related information were observed in selected groups, including hepatitis, mosquito-borne diseases, and sexually transmitted diseases.

Taken together, these analyses showed that suspicion-related information was distributed across multiple EHR document types and that the relative contribution of each source varied based on both the healthcare setting and the disease group.

## Discussion

This study highlights the obstacles in the current semimanual reporting methodology that render it suboptimal. An “overnotification” phenomenon was observed, with around 25% of wrongly reported cases in the sample. The most frequent cause for the mistake was a misinterpretation of terms related to the notifiable diseases. Although most patients are notified within the first few days of suspicion, there is still a delay of over 2 weeks in 10% of them, which, despite not representing a significant proportion of the cohort, reveals a problem that must be addressed. In contrast, a small number of episodes were reported before there was suspicion in the EHR. Probably, these episodes showcase the presence of not formal or registered communication channels such as phone calls or internal text messaging between teams before recording in the EHR. However, the source of the notification was not registered, and these explanations are hypotheses rather than established hypotheses. In addition, deriving external registered information outside the hospital EHR from other healthcare centers could have motivated suspicion. This highlights the importance of team building when designing the digital transformation of a process, as optimal human communication and strong IT systems are synergic.

With regard to the finding of thesaurus terms in the predefined documents, their presence varies based on the healthcare area and the disease group. The most prevalent ones in the ER were the ER discharge notes and the unreleased clinical drafts. In the hospital, the most prevalent ones were the hospital discharge notes, the unreleased clinical drafts, the clinical course reports, and the microbiology reports. Finally, in the outpatient clinic, there was a clear predominance of clinical course reports—the main record platform of consultations in this area. On a less important level, the laboratory and microbiology reports were also useful for suspicion in this area. Across disease groups, the ER discharge notes were more relevant in clinically diagnosed diseases, which are easily recognizable in an emergency setting, whereas hospital discharge notes gained importance in respiratory infections. The last group comprises legionellosis and tuberculosis. Both entities, when diagnosed in hospital services, usually involve pulmonary lesions or constitutional syndromes in patients with significant medical history. Hence, ER-diagnosed patients tend to be admitted for treatment and further diagnostic studies. Consequently, both legionellosis and tuberculosis are present in the hospital discharge notes. In contrast, this is probably not the best document to rely on as it only becomes available at the end of admission. Nonetheless, hospital discharge notes could be of interest for inclusion in a pipeline to identify patients whose clinical suspicion started after admission. The unreleased clinical draft is an unstructured and dynamic document and sometimes bears less specific information. Nonetheless, the value of this document lies in the fact that it appears at an early stage of the episode, contributing to faster identification, especially in disease groups with specific symptoms. The clinical course report visibly is the most representative of all, providing a variable degree of suspicion in all the analyzed disease groups. This is probably attributable to the fact that it captures the full trajectory of the condition of the patient throughout the duration for which they receive medical attention, with a relatively frequent and immediate record entry. Regarding this, the most frequently notified group were sexually transmitted diseases, with a 25.8%. In this hospital, STDs are mainly managed in the outpatient clinic, as they are usually addressed there from the ER. Even though, this workflow for STDs is probably not transferable to other institutions, where patients are handled in other healthcare areas. This showcases the importance of identifying hospital care pathways prior to training and adjusting AI algorithms. The laboratory and microbiology reports are representative to a variable degree, depending on the specific disease and its management. However, they are highly trustworthy when they provide a suspicious term because it usually confirms the disease. In the case of the “hepatitis” group, whose first suspicion usually includes an anomaly of biochemical parameters, there is a lack of representativity of this particular document. Upon closer inspection, the reason could be that these cases are frequently derived from external healthcare providers that made the first disease determination.

Importantly, these findings also provide information on the requirements of a future automated surveillance approach. The fact that disease-related terms were present in 83% of inadequate notifications indicates that simple keyword detection would be insufficient for this task. Future systems would need to distinguish active suspicion from historical diagnoses, chronic conditions, and contextually unrelated mentions, incorporating temporality and clinical context into their interpretation. Similarly, the marked differences in document relevance according to the healthcare setting and the disease group suggest that a future system should integrate multiple document types rather than relying on a single source, while also potentially prioritizing different documents according to the clinical pathway and the disease being assessed.

Based on these findings, several design considerations can be proposed for subsequent NLP development: selection and prioritization of document sources according to the disease and the healthcare setting; definition of standardized clinical concepts representing disease suspicion; contextual interpretation of clinical concepts rather than isolated keyword detection; and incorporation of expert validation within a human-in-the-loop surveillance workflow. These considerations are intended to inform subsequent system development rather than represent an NLP pipeline evaluated in the present study.

There is also another complementary approach that could support this process and came up after this initiation of this study. Since April 2024, Hospital Clínic de Barcelona has implemented a health problem list coded with SNOMED CT for all patients attended in the outpatient, inpatient, and ER environments. This problem list is entered by physicians when they want to document a patient's clinical scenario and whenever they create certain documents or enter a computerized provider order entry. This implementation provides real-time, standardized clinical concepts, which could also strengthen the early detection of entities that make up the timely suspicion for notifiable diseases.

Beyond traditional NLP approaches, recent advances in large language models (LLMs) offer a promising avenue for further optimizing the automated detection of notifiable diseases. Unlike conventional NLP methods that rely on predefined terminologies and structured term mappings, LLMs can process clinical text with a deeper contextual understanding (19). They can not only extract individual clinical entities (e.g., “fever” and “hemoptysis”) but also infer that their co-occurrence suggests a specific disease (e.g., tuberculosis). This ability to interpret symptoms, diagnoses, and risk factors in a more human-like manner could significantly enhance the sensitivity and specificity in automated reporting. Building on the insights from this study—particularly regarding the most informative clinical documents—we are currently implementing and evaluating LLMs for this task. This ongoing work aims to assess their performance in recognizing and classifying disease suspicions, with the goal of developing a more robust, scalable, and efficient reporting system. However, no NLP- or LLM-based system was evaluated in the present study, and therefore, no conclusions regarding their diagnostic or surveillance performance can be drawn from our results.

It is important to note again that, during the reporting process, external information sources might also be useful. However, these are not accessible from the hospital's EHR platform. This points to the need of a larger interconnectivity between the informatics services of all healthcare providers, which would also be beneficial for the improvement of an automatized reporting process. Access to previous reports would also be of interest to avoid repetitive notifications.

Nonetheless, several limitations in this study should be noted, including the sample size and duration of this study and the inclusion of only the 25 notifiable conditions reported during a 3-month summer period, which may limit the representativeness of the disease spectrum, as conditions with a different seasonal pattern may be underrepresented. Moreover, it does not count with information on previous reports, and therefore, the repetitive notification in chronic diseases could not be corroborated. For future studies, the inclusion of episodes that were both reported and not reported—identified through independent sources such as microbiology results or coded diagnoses—to have a notifiable condition could also be of interest to precisely determine the accuracy, sensitivity, and specificity of the reporting method. A more individualistic approach to each disease could also be made when addressing each document's representativity. Although the source clinical documentation was reviewed without preprocessing, identification and interpretation of suspicion terms were performed manually by investigators. Therefore, the present study characterizes the information available in raw EHR documentation but does not establish the performance that an automated NLP system would achieve on the same unprocessed text. Future studies should directly evaluate automated approaches on raw clinical documentation, including abbreviations, misspellings, colloquialisms, negation, temporality, and other sources of contextual and linguistic variability. Finally, larger datasets should be included in further research aimed at gathering more terms to allow an accurate terminological extraction, as the amount of training data is crucial to ensure an appropriate performance (20). Refining semantic annotation processes by leveraging the compiled thesaurus (nuancing context-dependent terms, adding new ones, and standardizing them) could contribute to the process. One of the main limitations of machine learning processes in the medical domain is the lack of clinical documents in many cases of patients due to privacy concerns (20, 21). On this note, pretraining models have demonstrated a powerful support for NLP (22), and the recent advent of locally trained generative pretrained transformers (GPTs) could allow the training without compromising data privacy.

## Conclusions

In summary, this study identified limitations in the timeliness and adequacy of the current semimanual notifiable disease–reporting process and characterized the EHR documents in which information supporting early disease suspicion is documented. The distribution of these signals varied substantially across healthcare settings and disease groups, while disease-related terms were also frequently present in inadequate notifications, underscoring the importance of contextual interpretation rather than simple keyword detection. These findings provide an empirical basis for the design of future NLP-assisted surveillance systems integrating multiple EHR document types and expert validation. However, no automated NLP system was evaluated in the present study, and subsequent development and validation on raw clinical documentation will be required to determine whether such approaches improve the accuracy and timeliness of notifiable disease reporting.

## Data Availability

The data analyzed in this study are subject to the following licenses/restrictions: data access can be requested via email to the corresponding author and is subject to approval by the Institutional Review Board. Requests to access these datasets should be directed to frid@clinic.cat.
