# Supplementary material for: Setting the basis for a natural language processing tool to improve the reporting of notifiable diseases: a descriptive study
Source: Front Digit Health. 2026 Oct 20;8:1882377. doi: 10.3389/fdgth.2026.1882377 (PMC13727731; doi:10.3389/fdgth.2026.1882377)
Supplement: Supplementary file 1 [file Table1.docx]

**Supplementary Table S1.** Compilation of the terms included in the standardized Thesaurus, classified per Notifiable Disease. Terms included are in Catalan, lowercase and without accents and translated in the corresponding column.

| **Notifiable disease** | **Standardized suspicion concepts** | **Concepts translation** |
| --- | --- | --- |
| Asthma | tos\; dispnea\; asma\; exacerbacio\; inhaladors\; sibilants\; expectoracio\; aguditzacio\; astenia\; hiperreactivitat bronquial\; hipoxemia\; febre\; taquipnea\; broncospasme\; crisi asmatica\; us musculatura accesoria\; tiratge\; febricula\; bronquitis asmatica | cough  dyspnea  asthma  exacerbation  inhalers  wheezing  sputum  aggravation  asthenia  bronchial hyperreactivity  hypoxemia  fever  tachypnea  bronchospasm  asthma attack  use of accessory muscles  retractions  mild fever  asthmatic bronchitis |
| Chickenpox | exantema cefalocaudal\; pruija \; infeccio per virus varicel·la\; varicel·la\; vvz\; eritema vesiculopustulos\; virus varicel·la-zoster positiu\; rash vesiculopapular\; meningitis limfocitaria vvz\; febre\; ac igm varicel·la-zoster positiu | cephalocaudal exanthem\  pruritus\  varicella virus infection\  varicella\  VZV\  vesiculopustular erythema\  positive varicella-zoster virus test\  vesiculopapular rash\  VZV lymphocytic meningitis\  fever\  positive varicella-zoster IgM antibodies |
| **Notifiable disease** | **Standardized suspicion concepts** | **Concepts translation** |
| Parotiditis | augment de volum regio parotidia\; edema parotidi\; febre\; dolor masticacio\; dolor parotidi\; parotiditis aguda\; igg v. parotiditis positiu\; pcr virus de la parotiditis\; tumefaccio parotidia\; parotiditis | enlargement of the parotid region\  parotid edema\  fever\  pain on mastication\  parotid pain\  acute parotitis\  positive mumps virus IgG\  mumps virus PCR\  parotid swelling\  parotitis |
| Shigellosis | febre\; diarrea aquosa\; astenia\; shigella\; shigella spp positiu\; diarrea\; vomits\; febricula\; colitis\; dolor abdominal\; shigella positiu\; gastroenteritis per shigella | fever\  watery diarrhea\  asthenia\  Shigella\  positive Shigella spp. test\  diarrhea\  vomiting\  low-grade fever\  colitis\  abdominal pain\  positive Shigella test\  Shigella gastroenteritis |
| Typhoid and paratyphoid fever | viatge\; dolor abdominal\; febre tifoide\; salmonella\; febre\; diarrea\; malestar general\; cefalea\; salmonella typhi | travel\  abdominal pain\  typhoid fever\  Salmonella\  fever\  diarrhea\  general malaise\  headache\  Salmonella Typhi |
| **Notifiable disease** | **Standardized suspicion concepts** | **Concepts translation** |
| Heatstroke | cop de calor\; vomits\; diarrea\; malestar general\; esforç fisic\; exposicio al sol\; esvaiment\; lipotimia\; insolacio\; exposicio a alta temperatura\; mareig | heatstroke\  vomiting\  diarrhea\  general malaise\  physical exertion\  sun exposure\  fainting\  presyncope\  sunstroke\  exposure to high temperatures\  dizziness |
| Hepatitis B | vhb\; hbsag\; hepatitis b\; hbe\; anti hbc positiu\; antigen superficie\; hbsag positiu\; igg antigen vhb positiu\; dna vhb positiu\; anti hbe positiu\; vhb positiu\; hbv comsava\; igg antigen core positiu\; igm antigen core positiu | HBV\  HBsAg\  hepatitis B\  HBe antigen\  positive anti-HBc antibodies\  surface antigen\  positive HBsAg\  positive HBV antigen IgG\  positive HBV DNA\  positive anti-HBe antibodies\  positive HBV test\  HBV ComSava\  positive core antigen IgG\  positive core antigen IgM |
| Hepatitis C | vhc\; hepatitis c\; arn vhc positiu\; vhc positiu\; anti vhc positiu\; reinfeccio\; arn vhc\; Hcvhc | HCV\  hepatitis C\  positive HCV RNA\  positive HCV test\  positive anti-HCV antibodies\  reinfection\  HCV RNA\  HCV |
| **Notifiable disease** | **Standardized suspicion concepts** | **Concepts translation** |
| Meningococcal disease | febre\; cefalea\; meningitis\; neisseria meningitidis\; meningoencefal·litis\; vomits | fever\  headache\  meningitis\  Neisseria meningitidis\  meningoencephalitis\  vomiting |
| Chikungunya | chikungunya\; artralgia\; astenia | chikungunya\  arthralgia\  asthenia |
| Chikungunya virus disease | febre\; artralgia\; chikungunya\; pcr virus chikungunya\; cefalea\; igg virus chikungunya positiu\; igm virus chikungunya positiu | fever\  arthralgia\  chikungunya\  chikungunya virus PCR\  headache\  positive chikungunya virus IgG\  positive chikungunya virus IgM |
| Dengue | febre\; artromialgia\; erupcio cutania\; denv\; arbovirus\; trombocitopenia\; viatge\; dengue\; leucopenia\; diarrea\; cefalea\; dolor retrorbitari\; mialgia\; calfreds\; anemia\; gota grossa\; falciparum\; plasmodium\; malaria\; artralgia\; antigen ns1 dengue positiu\; pcr virus dengue positiu\; Chikungunya | fever\  arthromyalgia\  skin rash\  DENV\  arbovirus\  thrombocytopenia\  travel\  dengue\  leukopenia\  diarrhea\  headache\  retro-orbital pain\  myalgia\  chills\  anemia\  thick blood smear\  P. falciparum\  Plasmodium\  malaria\  arthralgia\  positive dengue NS1 antigen\  positive dengue virus PCR\  chikungunya |
| **Notifiable disease** | **Standardized suspicion concepts** | **Concepts translation** |
| Malaria | paludisme\; febre\; anemia\; gota grossa\; falciparum\; ag plasmodium positiu\; plasmodium falciparum\; malaria\; trofozoits\; parasitemia\; p. falciparum\; viatge\; p. vivax\; plasmodium vivax\; gametocists\; calfreds\; ictericia\; astenia\; cefalea | malaria\  fever\  anemia\  thick blood smear\  P. falciparum\  positive Plasmodium antigen\  Plasmodium falciparum\  malaria\  trophozoites\  parasitemia\  P. falciparum\  travel\  P. vivax\  Plasmodium vivax\  gametocytes\  chills\  jaundice\  asthenia\  headache |
| Zika | viatge\; febre\; calfreds\; igm zika positiu\; zika | travel\  fever\  chills\  positive Zika IgM\  Zika |
| Hydatidosis | quist hidatidic\; equinococosi\; hidatidosi\; equinococ \; equinococcus | hydatid cyst\  echinococcosis\  hydatidosis\  echinococcus\  Echinococcus |
| Leishmaniasis | leishmaniosi cutania\; leishmaniosi\; leishmaniosi visceral\; leishmania | cutaneous leishmaniasis\  leishmaniasis\  visceral leishmaniasis\  Leishmania |
| Leptospirosis | leptospira\; espiroquetes\; febre\; artromialgia\; cefalea | Leptospira\  spirochetes\  fever\  arthromyalgia\  headache |
| Other acute respiratory | - | - |
| **Notifiable disease** | **Standardized suspicion concepts** | **Concepts translation** |
| Legionellosis | febre\; calfreds\; legionella pneumophila\; pneumonia\; legionel·losi\; pneumonia per legionella\; legionella\; dispnea | fever\  chills\  Legionella pneumophila\  pneumonia\  legionellosis\  Legionella pneumonia\  Legionella\  dyspnea |
| Tuberculosis | micobacteris\; perdua de pes\; tos\; dispnea\; anorexia\; astenia\; febre\; sudoracio nocturna\; contacte tuberculosi\; tuberculosi\; m. tuberculosis\; tbc pulmonar activa\; expectoracio hemoptoica\; tbc \; tbc\; cavitacio\; cavitacio tuberculosa\; tuberculoma\; quantifero positiu\; baar\; tbc ganglionar\; granuloma\; mal de pott\; tbc pulmonar\; tuberculosi disseminada\; ppd positiva\; pleuritis tuberculosa\; tuberculosi pulmonar\; tuberculosi pleural\; caverna\; tuberculosi peritoneal | mycobacteria\  weight loss\  cough\  dyspnea\  anorexia\  asthenia\  fever\  night sweats\  tuberculosis contact\  tuberculosis\  M. tuberculosis\  active pulmonary tuberculosis\  hemoptoic sputum\  TB\  TB\  cavitation\  tuberculous cavitation\  tuberculoma\  positive QuantiFERON test\  acid-fast bacilli\  lymph node tuberculosis\  granuloma\  Pott disease\  pulmonary tuberculosis\  disseminated tuberculosis\  positive PPD test\  tuberculous pleuritis\  pulmonary tuberculosis\  pleural tuberculosis\  cavity\  peritoneal tuberculosis |
| **Notifiable disease** | **Standardized suspicion concepts** | **Concepts translation** |
| Acquired immunodeficiency syndrome (AIDS) | sida\; infeccio per vih en estadi de sida\; wasting syndrome\; anticossos anti vih positius\; antigen p24 positiu\; carrega viral vih 1\; igg vih positiu\; vih | AIDS\  HIV infection at the AIDS stage\  wasting syndrome\  positive anti-HIV antibodies\  positive p24 antigen\  HIV-1 viral load\  positive HIV IgG\  HIV |
| Chlamydia genital infection | disuria\; uretritis\; ct\; contacte sexual de risc\; csr\; chlamydia\; doxiciclina\; chlamydia trachomatis\; clamidia | dysuria\  urethritis\  CT\  high-risk sexual contact\  high-risk sexual contact\  Chlamydia\  doxycycline\  Chlamydia trachomatis\  chlamydia |
| Gonococcal infection | contacte sexual de risc\; pcr neisseria gonorrhoeae \; csr\; secrecio uretral blanquinosa\; pruija uretral\; neisseria gonorrhoeae\; eritema penea\; balanitis\; gonorrea\; pcr neisseria gonorrhoeae\; ng \; pcr neisseria gonorrhoeae positiu\; uretritis\; ng\; proctitis\; disuria\; gonococ\; proctocolitis\; faringitis\; n. ghonorrea\; uretritis gonococcica\; tacte rectal doloros\; artritis gonococcica\; lesions vesiculo-pustuloses a palmells i eeii\; secrecio uretral groguenca\; relacio sexual sense proteccio\; alteracio flux vaginal\; leucorrea\; adenopaties inguinals\; salpingitis\; test gonorrea positiu\; eritema escrotal\; secrecio uretral | high-risk sexual contact\  Neisseria gonorrhoeae PCR\  high-risk sexual contact\  whitish urethral discharge\  urethral pruritus\  Neisseria gonorrhoeae\  penile erythema\  balanitis\  gonorrhea\  Neisseria gonorrhoeae PCR\  NG\  positive Neisseria gonorrhoeae PCR\  urethritis\  NG\  proctitis\  dysuria\  gonococcus\  proctocolitis\  pharyngitis\  N. gonorrhoeae\  gonococcal urethritis\  painful digital rectal examination\  gonococcal arthritis\  vesiculopustular lesions on the palms and lower extremities\  yellowish urethral discharge\  unprotected sexual intercourse\  altered vaginal discharge\  leukorrhea\  inguinal lymphadenopathy\  salpingitis\  positive gonorrhea test\  scrotal erythema\  urethral discharge |
| HIV infection | infeccio per vih\; carrega viral vih 1\; serologia vih positiva\; anticossos anti vih positius\; antigen p24 positiu\; contacte sexual de risc\; igg vih positiu\; malaltia pel virus de la immunodeficiencia humana\; vih\; tarv | HIV infection\  HIV-1 viral load\  positive HIV serology\  positive anti-HIV antibodies\  positive p24 antigen\  high-risk sexual contact\  positive HIV IgG\  human immunodeficiency virus disease\  HIV\  antiretroviral therapy |
| **Notifiable disease** | **Standardized suspicion concepts** | **Concepts translation** |
| Syphilis | sifilis\; sifilis latent tardana\; igg t. pallidum positiu\; prova reaginica lues\; vdrl\; serologia luetica positiva\; tpha positiu\; clia positiu\; igm prova treponemica positiu\; lues\; exantema maculopapular\; sifilis secundaria\; serologia treponemica positiva\; exantema\; csr\; contacte sexual de risc\; treponema pallidum\; xancre sifilitic\; secundarisme sifilitic\; rpr\; lesio ulcerativa indolora genital\; lesio crostrosa genital\; adenopaties inguinals\; sifilis primaria\; sifilis precoç\; secundarisme luetic\; lesions no pruriginoses perianals\; lesions no pruriginoses genitals\; lesions no pruriginoses difuses\; sifilis precoç primaria\; tp \; treponema positiu \; ulcera rectal | syphilis\  late latent syphilis\  positive T. pallidum IgG\  nontreponemal test for syphilis\  VDRL\  positive syphilis serology\  positive TPHA\  positive CLIA\  positive treponemal IgM test\  syphilis\  maculopapular exanthem\  secondary syphilis\  positive treponemal serology\  exanthem\  high-risk sexual contact\  high-risk sexual contact\  Treponema pallidum\  syphilitic chancre\  secondary syphilis\  RPR\  painless genital ulcerative lesion\  crusted genital lesion\  inguinal lymphadenopathy\  primary syphilis\  early syphilis\  secondary syphilis\  nonpruritic perianal lesions\  nonpruritic genital lesions\  diffuse nonpruritic lesions\  early primary syphilis\  TP\  positive Treponema test\  rectal ulcer |
